# Biofilm formation is not an independent risk factor for mortality in patients with *Acinetobacter baumannii* bacteremia

**DOI:** 10.3389/fcimb.2022.964539

**Published:** 2022-09-16

**Authors:** Tsung-Ta Chiang, Tzu-Wen Huang, Jun-Ren Sun, Shu-Chen Kuo, Aristine Cheng, Chang-Pan Liu, Yuag-Meng Liu, Ya-Sung Yang, Te-Li Chen, Yi-Tzu Lee, Yung-Chih Wang

**Affiliations:** ^1^ Division of Infectious Diseases and Tropical Medicine, Department of Internal Medicine, Tri-Service General Hospital, National Defense Medical Center, Taipei, Taiwan; ^2^ Department of Microbiology and Immunology, School of Medicine, College of Medicine, Taipei Medical University, Taipei, Taiwan; ^3^ Graduate Institute of Medical Sciences, College of Medicine, Taipei Medical University, Taipei, Taiwan; ^4^ Institute of Preventive Medicine, National Defense Medical Center, Taipei, Taiwan; ^5^ National Institute of Infectious Diseases and Vaccinology, National Health Research Institute, Maoli County, Taiwan; ^6^ Department of Internal Medicine, National Taiwan University Hospital, Taipei, Taiwan; ^7^ College of Medicine, National Taiwan University, Taipei, Taiwan; ^8^ Division of Infectious Diseases, Department of Internal Medicine, Mackay Memorial Hospital, Taipei, Taiwan; ^9^ Department of Medical Research, Mackay Memorial Hospital, Taipei, Taiwan; ^10^ Division of Infectious Diseases, Department of Internal Medicine, Changhua Christian Hospital, Changhua, Taiwan; ^11^ Graduate Institute of Life Sciences, National Defense Medical Center, Taipei, Taiwan; ^12^ Department of Emergency Medicine, Taipei Veterans General Hospital, Taipei, Taiwan; ^13^ Faculty of Medicine, School of Medicine, National Yang-Ming University, Taipei, Taiwan

**Keywords:** *Acinetobacter baumannii*, bacteremia, biofilm, carbapenem resistance, mortality

## Abstract

In the past decades, due to the high prevalence of the antibiotic-resistant isolates of *Acinetobacter baumannii*, it has emerged as one of the most troublesome pathogens threatening the global healthcare system. Furthermore, this pathogen has the ability to form biofilms, which is another effective mechanism by which it survives in the presence of antibiotics. However, the clinical impact of biofilm-forming *A. baumannii* isolates on patients with bacteremia is largely unknown. This retrospective study was conducted at five medical centers in Taiwan over a 9-year period. A total of 252 and 459 patients with bacteremia caused by biofilm- and non-biofilm-forming isolates of *A. baumannii*, respectively, were enrolled. The clinical demographics, antimicrobial susceptibility, biofilm-forming ability, and patient clinical outcomes were analyzed. The biofilm-forming ability of the isolates was assessed using a microtiter plate assay. Multivariate analysis revealed the higher APACHE II score, shock status, lack of appropriate antimicrobial therapy, and carbapenem resistance of the infected strain were independent risk factors of 28-day mortality in the patients with *A. baumannii* bacteremia. However, there was no significant difference between the 28-day survival and non-survival groups, in terms of the biofilm forming ability. Compared to the patients infected with non-biofilm-forming isolates, those infected with biofilm-forming isolates had a lower in-hospital mortality rate. Patients with either congestive heart failure, underlying hematological malignancy, or chemotherapy recipients were more likely to become infected with the biofilm-forming isolates. Multivariate analysis showed congestive heart failure was an independent risk factor of infection with biofilm-forming isolates, while those with arterial lines tended to be infected with non-biofilm-forming isolates. There were no significant differences in the sources of infection between the biofilm-forming and non-biofilm-forming isolate groups. Carbapenem susceptibility was also similar between these groups. In conclusion, the patients infected with the biofilm-forming isolates of the *A. baumannii* exhibited different clinical features than those infected with non-biofilm-forming isolates. The biofilm-forming ability of *A. baumannii* may also influence the antibiotic susceptibility of its isolates. However, it was not an independent risk factor for a 28-day mortality in the patients with bacteremia.

## Introduction

*Acinetobacter baumannii* is an important pathogen responsible for various nosocomial infections and leads to high rates of mortality and morbidity in the infected patients (Cisneros et al., 1996; Dijkshoorn et al., 2007). Its emergence as a drug-resistant pathogen has made the treatment of infected patients difficult (Dijkshoorn et al., 2007; Peleg et al., 2008). Moreover, *A. baumannii* has been found to have the ability to form biofilms, which is another effective way for the bacteria to survive in the presence of antibiotics *(* Villegas and Hartstein, 2003; Martí et al., 2011 *).* Biofilm, a three-dimensional structure constructed by the bacterial community, is encased in an extracellular polymeric matrix (Hall-Stoodley et al., 2004). Biofilms may act as a barrier against the penetration of antimicrobials, to alter their metabolism and effects, resulting in antimicrobial resistance (Harrison et al., 2007). However, some studies have shown that biofilm-forming isolates exhibit variations in drug resistance (Rodríguez-Baño et al., 2008; Qi et al., 2016; Donadu et al., 2021). These findings suggest that the biofilm-forming ability of bacterial isolates may contribute to differences in drug resistance. Moreover, biofilm- and non-biofilm-forming isolates could show different virulence.

*A. baumannii* can cause various biofilm-associated infections, such as chronic wound infections, ventilator-associated pneumonia, infective endocarditis, and catheter-related infections (Høiby et al., 2015; Gedefie et al., 2021). Although biofilm-associated infections are considered the cause of morbidity and mortality in patients, several studies have reported that infections caused by them are not associated with worse outcomes (Rodríguez-Baño et al., 2008; Barsoumian et al., 2015; Wang et al., 2018). Therefore, the clinical impact of the biofilm-forming ability of the pathogen remains elusive. In previous studies, we found that biofilm formation was not associated with worse outcomes in *A. baumannii* bacteremic pneumonia (Wang et al., 2018). However, different types of biofilm-associated infections may result in different clinical outcomes. Limited data are available regarding the clinical impacts of biofilm-forming ability of the isolates of *A. baumannii*. This study aimed to establish a correlation between the biofilm-forming ability of *A. baumannii* and the clinical outcomes in patients with *A. baumannii* bacteremia.

## Materials and methods

### Hospital setting and study population

This retrospective study was conducted from January 2010 to December 2019 at five medical centers in Taiwan, namely (alphabetically) Changhua Christian Hospital (CCH, 1676 beds) in Central Taiwan, Mackay Memorial Hospital (MMH, 2055 beds), National Taiwan University Hospital (NTUH, 2245 beds), Taipei Veterans General Hospital (TVGH, 2900 beds), and Tri-Service General Hospital (TSGH, 1712 beds) of National Defense Medical Center in Northern Taiwan. Patients who had at least one positive blood culture for *A. baumannii* and who simultaneously had symptoms and signs of infection were enrolled. In patients with two or more positive blood cultures, only the first blood culture was included. Patients under 20 years of age and with incomplete medical records were excluded. The study protocol was approved by the institutional review board (IRB) of each hospital (approval numbers: CCH: IRB No. 140514, MKH: IRB No. 14MMHIS125, NTU: IRB No. 201008047R, TSGH: IRB No. 1-103-05-100, and TVGH: IRB No. 2015-04-003C).

An episode of *A. baumannii* bacteremia was defined as the isolation of *A. baumannii* from a blood culture on one or more occasions. The onset of bacteremia was defined as the day when the blood culture that eventually yielded *A. baumannii* was obtained. Bacteremia episodes in the intensive care unit (ICU) were defined as having occurred within 48 h of ICU admission. A previous stay in the ICU was defined as admission to the ICU within 30 days prior to the bacteremia onset. Previous use of antimicrobials was defined as the use of antimicrobials, 30 days preceding the date of bacteremia onset. Those who received immunosuppressant agents within 2 weeks or corticosteroids at a dosage equivalent to or higher than 15 mg of prednisolone daily for 1 week within 4 weeks prior to the bacteremia onset were considered to have received immunosuppressant therapy. Chemotherapy use was defined as the administration of cytotoxic agents within 6 weeks prior to the onset of bacteremia. Recent surgery was defined as a surgery performed within 4 weeks prior to the onset of bacteremia. The source of bacteremia was determined according to the US Centers for Disease Control and Prevention definitions (Garner et al., 1988). The Acute Physiology and Chronic Health Evaluation (APACHE) II score within 24 h prior to the bacteremia onset was used to assess the severity of the disease. The all-cause 28-day mortality was defined as death occurring within 28 days of the onset of bacteremia and was set as the endpoint. The survival status of those who were discharged before the 28-day period was determined by contacting the patient or reviewing their medical records. None of the patients in this group were lost to follow-up.

### Bacterial identification and antimicrobial susceptibility testing

Presumptive identification of the isolates at the *A. baumannii* complex (Abc) level was performed using the Vitek 2 system (bioMérieux, Marcy l’Etoile, France). The multiplex polymerase chain reaction was carried out to identify *A. baumannii* at the level of genomic species (Chen et al., 2007). The minimum inhibitory concentrations (MICs) of antimicrobial agents were determined by broth microdilution (Wayne and CLSI., 2006) and interpreted according to the standards given by the Clinical and Laboratory Standards Institute (CLSI) standards (Wayne and CLSI., 2020).

### Biofilm cultivation and measurement

Biofilm-forming capability was quantitatively estimated using the crystal violet staining method (O’Toole et al., 1999; Wang et al., 2016). However, minor modifications in the procedure were made. Briefly, the bacterial strains were cultured at 37°C for 24 h in 5 mL Luria-Bertani (LB) broth supplemented with 1% D–glucose (LBglu). The cultures were diluted in LBglu to achieve an optical density (OD) of 0.03 at a wavelength of 570 nm. Aliquots of 200 μL of the final solution were added to each well of a 96-well tissue culture polystyrene microtiter plate. After incubation with agitation for 48 h at 37°C, the suspensions were removed and the wells were washed with phosphate-buffered saline (PBS), followed by the addition of 200 μL of 0.1% crystal violet to stain the cells. The plates were then incubated for 20 min with gentle agitation and washed. The crystal violet of the stained biofilms were solubilized with 200 μL of 95% ethanol for 10 min with agitation. The amount of biofilm formed was quantified by measuring the optical density at 570 nm (OD_570_). All experiments were performed in triplicates and repeated on three separate occasions. The OD_570_ values of the well with un-inoculated LB medium were used as a negative control. Those with OD_570_ values at least twice that of the negative controls on at least two separate occasions were considered as biofilm formation positive (Rodríguez-Baño et al., 2008).

### Statistical analyses

The data were analyzed using the statistical package PASW for the Windows version 26 (SPSS, Chicago, IL, USA). The χ^2^ test with Yate’s correction or Fisher’s exact test was used to compare the categorical differences. Continuous variables were analyzed using the Student’s *t* test and data were presented as median and interquartile range (IQR). The time to mortality, defined as the interval between bacteremia onset and death, was analyzed using Kaplan–Meier survival analysis, and log-rank test was used to compare the univariate survival distribution between different groups of patients. The logistic regression model was used to explore independent prognostic factors associated with the 28-day mortality. Univariate analyses were performed for each of the risk factors to ascertain the odds ratio (OR) and 95% confidence interval (CI). All biologically plausible variables with a *P* < 0.10 in the univariate analysis were considered for inclusion in the multivariate logistic regression model with a backward selection process. A *P* < 0.05 was considered statistically significant.

## Results

Patients who had experienced at least one episode of the *A. baumannii* complex monomicrobial bacteremia during the 9-year period were enrolled for evaluation. Those with bacteremia caused by non-*baumannii Acinectobacter* spp. were excluded from the analysis. Ultimately, of 711 patients that were enrolled, 385 (54.15%) survived and 326 (45.85%) died within 28 days of the onset of *A. baumannii* bacteremia. The demographic and clinical features of the 28-days survivors and non-survivors are presented in **Table 1**. Multivariate logistic regression analysis was carried out (**Table 2**) to delineate the independent risk factors of 28-day mortality due to *A. baumannii* bacteremia. Previous exposure to fluoroquinolones (OR, 2.052; CI 1.182–3.564; *P* = 0.011), liver cirrhosis (OR, 2.395; CI, 1.196–4.796; *P* = 0.014), higher disease severity (APACHE II score) (OR, 1.147; CI, 1.116–1.178; *P* < 0.001), shock (OR, 1.863; CI, 1.179–2.944; *P* =0.008), receipt of a thoracic drain (OR, 5.502; CI, 1.889–16.021; *P* = 0.002), infection by carbapenem-resistant isolates (OR, 2.425; CI, 1.524–3.858; *P* < 0.001), and receipt of inappropriate antimicrobial therapy (OR, 1.670; CI, 1.051–2.655; *P* = 0.030) were independent risk factors of 28-day mortality. In contrast, those who had hypertension (OR, 0.523; CI, 0.336–0.815; *P* = 0.004), cerebrovascular disease (OR, 0.296; CI, 0.171–0.512; *P* < 0.001), underwent a surgery within the past 4 weeks (OR, 0.492; CI, 0.288–0.840; *P* = 0.009), and those who had developed bacteremia as a consequence of a urinary tract infection (OR, 0.381; CI, 0.161–0.904; *P* = 0.029) were more likely to survive the 28-days after developing bacteremia. The proportion of infection caused by biofilm formation isolates was not significant differed between survivors and non-survivors within 28 days. (38.4% *vs.* 31.9%, *P* = 0.071).

**Table 1:** Clinical characteristics and outcomes of patients with *Acinetobacter baumannii* bloodstream infections who survived or died within 28 days of bacteremia onset.

Variables	Survivors (*n* = 385)	Non-survivors (*n* =326)	*P* value
Demographic characteristics			
Age, median (IQR), years	71 (58–80)	72 (57–81)	0.722
Male sex, No. (%)	261 (67.8)	235 (72.1)	0.220
Acquired in ICU, No. (%)	151 (39.2)	179 (54.9)	< 0.001
Length of hospitalization before bacteremia, median (IQR), days	14 (5–32)	20 (9–38)	0.190
Previous use of antibiotics, No. (%)			
Aminoglycosides	41 (10.6)	27 (8.3)	0.308
Penicillins	47 (12.2)	37 (11.3)	0.816
β-lactam/β-lactamase inhibitors (except sulbactam)	43 (11.2)	46 (14.1)	0.256
Sulbactam	15 (3.9)	13 (4.0)	1.000
Non-anti-pseudomonas Cephalosporins	84 (21.8)	57 (17.5)	0.158
Anti-pseudomonas Cephalosporins	61 (15.8)	78 (23.9)	0.008
Group 2 carbapenems	61 (15.8)	71 (21.8)	0.043
Fluoroquinolones	41 (10.6)	83 (25.5)	< 0.001
Tigecycline	12 (3.1)	27 (8.3)	0.003
Colistin	7 (1.8)	18 (5.5)	0.013
Teicoplanin	53 (13.8)	72 (22.1)	0.004
Fluconazole	31 (8.1)	44 (13.5)	0.020
Comorbid condition, No. (%)			
Liver cirrhosis	27 (7.0)	47 (14.4)	0.002
Chronic obstructive pulmonary disease	62 (16.1)	66 (20.2)	0.170
Chronic kidney disease	110 (28.6)	111 (34.0)	0.123
Type 2 diabetes mellitus	125 (32.5)	95 (29.1)	0.371
Hypertension	158 (41.0)	104 (31.9)	0.013
Coronary artery disease	38 (9.9)	38 (11.7)	0.466
Congestive heart failure	53 (13.8)	54 (16.6)	0.344
Cerebrovascular accident	104 (27.0)	43 (13.2)	< 0.001
Collagen vascular disease	6 (1.6)	22 (6.7)	< 0.001
Immunosuppressant therapy	73 (19.0)	89 (27.3)	0.009
Solid tumor	74 (19.2)	62 (19.0)	1.000
Hematological malignancy	18 (4.7)	32 (9.8)	0.008
Chemotherapy	38 (9.9)	30 (9.2)	0.799
Trauma	19 (4.9)	5 (1.5)	0.012
Recent surgery	113 (29.4)	47 (14.4)	< 0.001
Previous ICU admission	179 (46.5)	190 (58.3)	0.002
Charlson comorbidity index, median (IQR)	3 (1–5)	3 (1–5)	0.279
APACHE II score, median (IQR)	18 (12–24)	29 (21–38)	< 0.001
Shock	76 (19.7)	153 (46.9)	< 0.001
Invasive Procedures, No. (%)			
Central venous catheter	139 (36.1)	144 (44.2)	0.031
Arterial line	67 (17.4)	80 (24.5)	0.020
Tracheostomy	41 (10.6)	33 (10.1)	0.902
Ventilator use	168 (43.6)	203 (62.3)	< 0.001
Hemodialysis	32 (8.3)	46 (14.1)	0.016
Thoracic drain	8 (2.1)	24 (7.4)	0.001
Abdominal drain	32 (8.3)	23 (7.1)	0.575
Total parental nutrition	23 (6.0)	27 (8.3)	0.242
Source of bacteremia, No. (%)			
Pneumonia	154 (40.0)	188 (57.7)	< 0.001
Catheter related bloodstream infection	63 (16.4)	47 (14.4)	0.533
Urinary tract infection	46 (11.9)	13 (4.0)	< 0.001
Intra-abdominal infection	35 (9.1)	23 (7.1)	0.339
Skin and soft tissue infection	22 (5.7)	12 (3.7)	0.222
Primary bacteremia	79 (20.5)	69 (21.2)	0.853
Surgical site infection	2 (0.5)	3 (0.9)	0.665
Central nerve system infection	3 (0.8)	2 (0.6)	1.000
Multisite infection	16 (4.2)	22 (6.7)	0.135
Biofilm formation, No. (%)	148 (38.4)	104 (31.9)	0.071
Carbapenem-resistance isolates	174 (45.2)	241 (73.9)	< 0.001
Inappropriate antimicrobial therapy, No. (%)	244 (63.4)	251 (77.0)	< 0.001

IQR, interquartile range; ICU, intensive care unit; APACHE II, Acute Physiology and Chronic Health Evaluation II.

**Table 2:** Logistic regression analysis for the risk of 28-day mortality in patients with *Acinetobacter baumannii* bloodstream infections.

Variables	Crude model			Model 1^*^			
		OR (95% CI)	*P*		OR (95% CI)		*P*	
Acquired in ICU	1.887 (1.399–2.545)	< 0.001		1.045 (0.659–1.656)		0.852	
Previous use of antibiotics							
Anti-pseudomonas Cephalosporins	1.671 (1.150–2.427)	0.007		1.511 (0.899–2.538)		0.119	
Group 2 carbapenems	1.479 (1.012–2.161)	0.043		0.914 (0.532–1.570)		0.745	
Fluoroquinolones	2.866 (1.905–4.312)	< 0.001		2.085 (1.182–3.564)		0.011	
Tigecycline	2.807 (1.3.98–5.634)	0.004		1.042 (0.414–2.624)		0.931	
Colistin	3.156 (1.301–7.653)	0.011		2.447 (0.779–7.687)		0.126	
Teicoplanin	1.776 (1.202–2.624)	0.004		1.120 (0.627–2.001)		0.701	
Fluconazole	1.782 (1.096–2.895)	0.020		0.670 (0.346–1.299)		0.236	
Comorbid condition							
Liver cirrhosis	2.234 (1.357–3.677)	0.002		2.395 (1.196–4.796)		0.014	
Hypertension	0.673 (0.494–0.917)	0.012		0.523 (0.336–0.815)		0.004	
Cerebrovascular accident	0.411 (0.277–0.607)	< 0.001		0.296 (0.171–0.512)		< 0.001	
Collagen vascular disease	4.571 (1.830–11.416)	0.001		2.729 (0.804–9.265)		0.108	
Immunosuppressant therapy	1.605 (1.128–2.283)	0.009		1.159 (0.678–1.955)		0.581	
Hematological malignancy	2.219 (1.221–4.034)	0.009		1.497 (0.627–3.572)		0.364	
Trauma	0.300 (0.111–0.813)	0.018		0.887 (0.273–2.877)		0.842	
Recent surgery	0.405 (0.278–0.592)	< 0.001		0.492 (0.288–0.840)		0.009	
Previous ICU admission	1.608 (1.194–2.165)	0.002		1.068 (0.642–1.777)		0.800	
APACHE II score, median (IQR)	1.135 (1.112–1.158)	< 0.001		1.147 (1.116–1.179)		< 0.001	
Shock	3.596 (2.580–5.012)	< 0.001		1.863 (1.179–2.944)		0.008	
Invasive Procedures, No. (%)							
Central venous catheter	1.400 (1.036–1.893)	0.029		0.753 (0.440–1.230)		0.241	
Arterial line	1.544 (1.072–2.223)	0.020		0.647 (0.348–1.204)		0.169	
Ventilator use	2.132 (1.577–2.881)	< 0.001		0.700 (0.423–1.160)		0.166	
Hemodialysis	1.812 (1.124–2.922)	0.015		0.900 (0.474–1.711)		0.748	
Thoracic drain	3.745 (1.659–8.455)	0.001		5.502 (1.889–16.021)		0.002	
Source of bacteremia							
Pneumonia	2.043 (1.514–2.758)	< 0.001		0.930 (0.594–1.458)		0.752	
Urinary tract infection	0.306 (0.162–0.577)	< 0.001		0.381 (0.161–0.904)		0.029	
Carbapenem-resistance isolates	3.438 (2.500–4.728)	< 0.001		2.425 (1.524–3.858)		< 0.001	
Inappropriate antimicrobial therapy	1.934 (1.389–2.693)	< 0.001		1.670 (1.051–2.655)		0.030	

OR, odds ratio; CI, confidence interval; ICU, intensive care unit; APACHE II, Acute Physiology and Chronic Health Evaluation II.^*^Adjusted by all factors included in the table.

**Figure 1:**
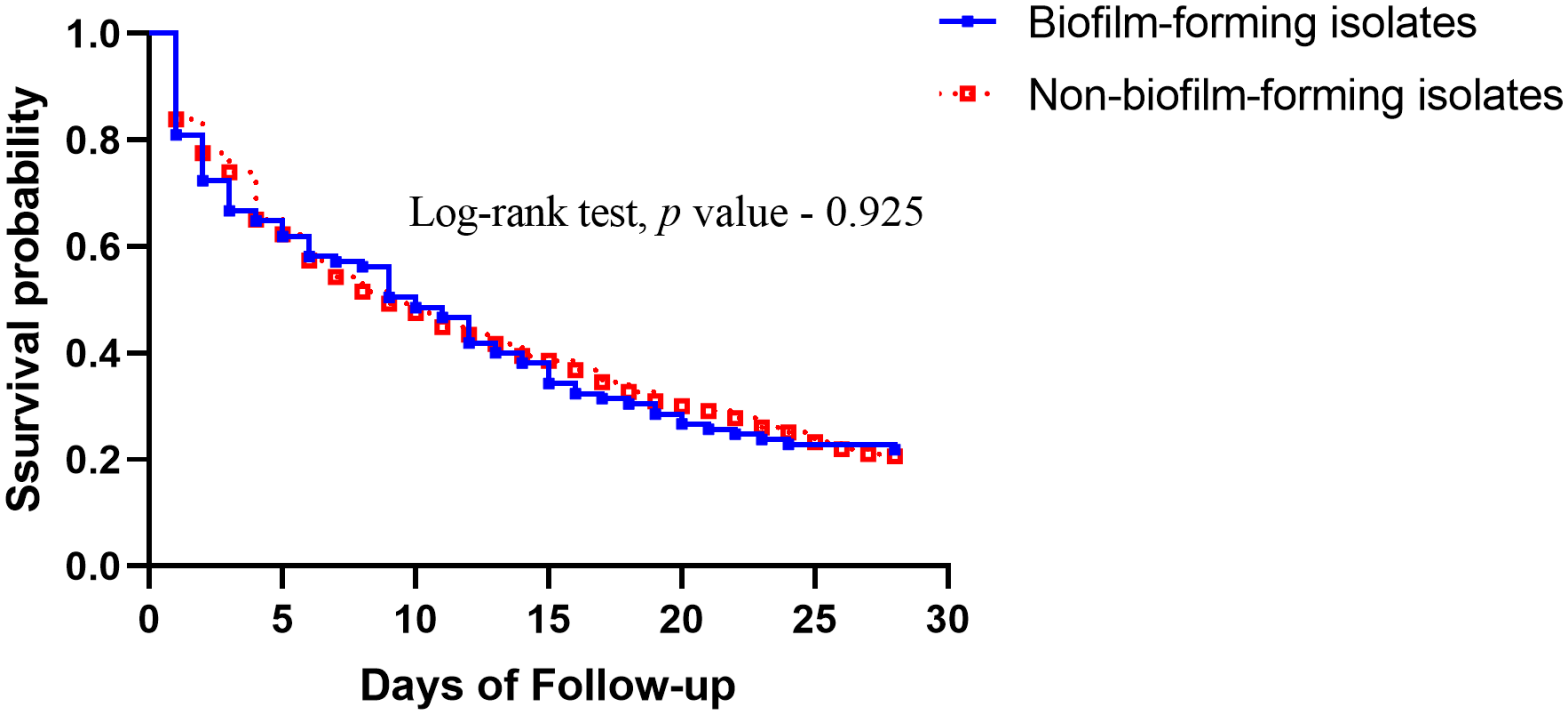
Comparison of Kaplan–Meier survival curves, at 28 days, between patients with *Acinetobacter baumannii* bacteremia caused by biofilm-forming isolates and non-biofilm-forming isolates.

To further explore the risk of the infection by biofilm-forming isolates, we divided the patients into two groups according to the biofilm-forming ability of the bacterial isolates. The demographic and clinical characteristics of patients with *A. baumannii* bacteremia caused by the biofilm-forming (n = 252, 35.44%) and non-biofilm-forming (n = 459, 64.56%) isolates are listed in **Table 3**. Those who became infected with biofilm-forming isolates were less likely to contract the infection in the ICU (40.1% *vs.* 49.9%, *P* = 0.015), more likely to have congestive heart failure (19.0% *vs.* 12.9%, *P* = 0.029), less likely to be exposed previously to penicillin (8.3% *vs.* 13.7%, *P*= 0.039), more likely to have hematological malignancy (10.3% *vs.* 5.2%, *P* = 0.014), more likely to receive chemotherapy (12.7% *vs.* 7.8%, *P* = 0.045), less likely to receive a central venous catheter insertion (28.2% *vs.* 46.2%, *P* < 0.001), less likely to receive an arterial line insertion (9.5% *vs.* 26.8%, *P* < 0.001), less likely to be on a ventilator (44% *vs.* 56.6%, *P* = 0.002), and less likely to receive a thoracic drain insertion (2% *vs.* 5.9%, *P* = 0.022). The resistance rates to carbapenems were similar for both biofilm-forming and non-biofilm-forming isolates (56.7% *vs.* 59.3%, *P* = 0.525). Furthermore, there was no significant difference in their 14-day and 28-day mortality rates (**Figure 1**). The overall mortality rate was higher in the non-biofilm-forming group by a borderline statistical difference, compared with the biofilm-forming group (58.4% *vs.* 50.4%, *P* = 0.048). Logistic regression analysis was performed to delineate the independent risk factors for the infection by the biofilm-forming isolates. As shown in **Table 4**, congestive heart failure was a risk factor of infection with biofilm-forming isolates (OR, 1.918; CI, 1.221–3.012; *P* = 0.005). However, those who received an arterial line were less likely to be infected with biofilm-forming isolates (OR, 0.416; CI, 0.240–0.721; *P* = 0.002).

**Table 3:** Clinical characteristics of patients and clinical isolates with biofilm-forming and non-biofilm-forming *Acinetobacter baumannii* blood stream infections.

	Biofilm-forming (*n* = 252)	Non-biofilm-forming (*n* = 459)	*P* value
Demographic characteristics			
Age, median (IQR), years	71 (57–81)	72 (57–80)	0.132
Male sex, No. (%)	171 (67.9)	325 (70.8)	0.443
Acquired in ICU, No. (%)	101 (40.1)	229 (49.9)	0.015
Length of hospitalization before bacteremia, median (IQR), days	16 (6.25–34)	18 (7–33)	0.231
Previous use of antibiotics, No. (%)			
Aminoglycosides	20 (7.9)	48 (10.5)	0.290
Penicillins	21 (8.3)	63 (13.7)	0.039
β-lactam/β-lactamase inhibitors (except sulbactam)	32 (12.7)	57 (12.4)	0.906
Sulbactam	11 (4.4)	17 (3.7)	0.689
Non-anti-pseudomonas Cephalosporins	46 (18.3)	95 (20.7)	0.491
Anti-pseudomonas Cephalosporins	52 (19.0)	87 (20.6)	0.621
Group 2 Carbapenems	38 (15.1)	94 (20.5)	0.087
Fluoroquinolones	39 (15.5)	85 (18.5)	0.353
Tigecycline	12 (4.8)	27 (5.9)	0.608
Colistin	6 (2.4)	19 (4.1)	0.289
Teicoplanin	36 (14.3)	89 (19.4)	0.099
Fluconazole	22 (8.7)	53 (11.5)	0.254
Comorbid condition, No. (%)			
Liver cirrhosis	27 (10.7)	47 (10.2)	0.898
Chronic obstructive pulmonary disease	40 (15.9)	88 (19.2)	0.308
Chronic kidney disease	84 (33.3)	137 (29.8)	0.352
Type 2 diabetes mellitus	74 (29.4)	146 (31.8)	0.553
Hypertension	87 (34.5)	175 (38.1)	0.372
Coronary artery disease	28 (11.1)	48 (10.5)	0.800
Congestive heart failure	48 (19.0)	59 (12.9)	0.029
Cerebrovascular accident	50 (19.8)	97 (21.1)	0.700
Collagen vascular disease	14 (5.6)	14 (3.1)	0.110
Immunosuppressant therapy	61 (24.2)	101 (22.0)	0.514
Solid tumor	57 (22.6)	79 (17.2)	0.090
Hematological malignancy	26 (10.3)	24 (5.2)	0.014
Chemotherapy	32 (12.7)	36 (7.8)	0.045
Recent surgery	48 (19.0)	112 (24.4)	0.111
Previous ICU admission	126 (50.0)	243 (52.9)	0.480
Charlson comorbidity index, median (IQR)	2 (1-5)	3 (2-6)	0.113
APACHE II score, median (IQR)	21 (14-29)	24 (17-31)	0.650
Shock, No. (%)	81 (32.1)	148 (32.2)	1.000
Invasive Procedures, No. (%)			
Central venous catheter	71 (28.2)	212 (46.2)	<0.001
Arterial line	24 (9.5)	123 (26.8)	<0.001
Tracheostomy	52 (11.3)	22 (8.7)	0.306
Ventilator use	111 (44.0)	260 (56.6)	0.002
Hemodialysis	20 (7.9)	58 (12.6)	0.060
Thoracic drain	5 (2.0)	27 (5.9)	0.022
Abdominal drain	13 (5.2)	42 (9.2)	0.058
Total parental nutrition	17 (6.7)	33 (7.2)	0.879
Carbapenem resistance, No. (%)	143 (56.7)	272 (59.3)	0.525
Appropriate antimicrobial therapy	87 (34.5)	129 (28.1)	0.088
Outcome			
14-day mortality, No. (%)	87 (34.5)	180 (39.2)	0.226
28-day mortality, No. (%)	104 (41.3)	222 (48.4)	0.071
Overall Mortality, No. (%)	127 (50.4)	268 (58.4)	0.048

IQR, interquartile range; ICU, intensive care unit; APACHE II, Acute Physiology and Chronic Health Evaluation II.

**Table 4:** Logistic regression analysis of predictors for patients infected with biofilm-forming *Acinetobacter baumannii*.

Characteristic	Univariate analysis		Multivariate analysis	
	Crude OR (95% CI)	*p*	Adjusted OR (95% CI)	*p*
Acquired in ICU	0.672 (0.492–0.917)	0.012		
Previous use of penicillins	0.571 (0.340–0.961)	0.035		
Previous use of group 2 Carbapenems	0.690 (0.456–1.042)	0.078		
Previous use of teicoplanin	0.693 (0.454–1.057)	0.088		
Congestive heart failure	1.595 (1.052–2.420)	0.028	1.918 (1.221–3.012)	0.005
Solid tumor	1.406 (0.960–2.060)	0.080		
Hematological malignancy	2.085 (1.170–3.715)	0.013		
Chemotherapy	1.709 (1.033–2.827)	0.037		
Central venous catheter	0.457 (0.329–0.636)	<0.001		
Arterial line	0.288 (0.180–0.459)	<0.001	0.416 (0.240–0.721)	0.002
Ventilator use	0.603 (0.442–0.821)	0.001		
Hemodialysis	0.596 (0.350–1.016)	0.057		
Thoracic drain	3.087 (1.174–8.120)	0.022		
Abdominal drain	0.540 (0.284–1.026)	0.060		

ICU, intensive care unit; CI, confidence interval; OR, odds ratio.

We further stratified the strains according to the infection foci that resulted in bacteremia (**Table 5**). Pneumonia was the most common source of infection, followed by primary bacteremia and catheter-related bloodstream infections. There were no significant difference in the sources of bacteremia between the biofilm-forming and non-biofilm-forming isolates.

**Table 5:** Types of infections caused by biofilm-forming and non-biofilm-forming isolates of *Acinetobacter baumannii*.

Sources of bacteremia	Biofilm-forming (*n* = 252)	Non-biofilm-forming (*n* = 459)	*P*-value	Overall Mortality	28-day Mortality
Pneumonia, No. (%)	111 (44.0)	231 (50.3)	0.117	67.8%	55.0%
Catheter related bloodstream infection, No. (%)	45 (17.9)	65 (14.2)	0.195	48.2%	42.7%
Urinary tract infection, No. (%)	25 (9.9)	34 (7.4)	0.257	28.8%	22.0%
Intra-abdominal infection, No. (%)	27 (10.7)	31 (6.8)	0.085	48.3%	39.7%
Skin and soft tissue infection, No. (%)	11 (4.4)	23 (5.0)	0.855	52.9%	35.3%
Primary bacteremia, No. (%)	56 (22.2)	92 (20.0)	0.500	52.0%	46.6%
Central nerve system infection, No. (%)	0 (0.0)	5 (1.1)	0.167	60.0%	40.0%
Surgical site infection, No. (%)	2 (0.8)	3 (0.7)	1.000	80.0%	60.0%
Multisite infection, No. (%)	19 (7.5)	19 (4.1)	0.057	71.1%	57.9%

A subgroup analysis was conducted to assess the risk factors of 28-day mortality in the patients infected with biofilm-forming isolates (**Supplementary Table S1**). Those who had developed bacteremia in the ICU, had previous exposure to fluoroquinolones, had collagen vascular disease, were recipients of immunosuppressant therapy, had hematological malignancy, had previous ICU admission history, were recipients of ventilator support, were infected with carbapenem-resistant isolates, had presented with shock, had higher APACHE II score, had bacteremia secondary to pneumonia, or had a multisite infection, were associated with a higher 28-day mortality rate. In contrast, those who underwent surgery within 4 weeks prior to the onset of bacteremia, those who had received appropriate antimicrobial therapy, those who had bacteremia secondary to urinary tract infection were associated with lower 28-day mortality rates. In the logistic regression analysis, those who had hematological malignancy (OR, 3.636; CI, 1.011–13.072; *P* = 0.048), infected with carbapenem-resistant isolates (OR, 2.945; CI, 1.344–6.453; *P* = 0.007), and had higher APACHE II score (OR, 1.151; CI, 1.098–1.206; *P* < 0.001) were independently associated with the 28-day mortality rates (**Supplementary Table S2**).

## Discussion

This study revealed that there were no significant differences in the 14-day and 28-day mortality rates between patients infected with the biofilm-forming and non-biofilm-forming isolates of *A. baumannii*. Previous exposure to fluoroquinolones, liver cirrhosis, higher APACHE II score, shock status, infection with carbapenem-resistant isolates, and receipt of inappropriate antimicrobial therapy were independent risk factors of 28-day mortality in patients with *A. baumannii* bacteremia. Congestive heart failure was an independent risk factor of infection with biofilm-forming isolates, while the patients with an arterial line were more likely to be infected with non-biofilm-forming isolates.

It is not surprising that shock status, higher APACHE II score, infection with carbapenem-resistant *A. baumannii*, and inappropriate treatment were independent risk factors for mortality, which is consistent with previous findings (Peleg et al., 2008; Lee et al., 2012; Wong et al., 2017). Those with liver cirrhosis had a higher 28-day mortality in this study. Our previous study also demonstrated a higher 30-day mortality rate in patients with liver cirrhosis compared to those without cirrhosis;, however, there was no significant difference (Liu et al., 2019). That study investigated all *Acinetobacter* species and enrolled a relatively small number of patients with *A. baumannii* bacteremia. The relatively small population of patients with *A. baumannii* bacteremia in that study may have contributed to the insignificance.

Patients with a previous exposure to fluoroquinolones had worse clinical outcomes. Although there is limited research regarding the correlation between fluoroquinolone exposure and outcomes in patients with *A. baumannii* bacteremia, one study concluded that exposure to fluoroquinolones is an independent risk factor for the development of carbapenem-resistant *A. baumannii* bacteremia (Kopterides et al., 2007). This may explain the risk of mortality in patients with *A. baumannii* bacteremia in our study.

The components of biofilms and their unique environment overpower most antimicrobials used for treating biofilm-associated infections (Høiby et al., 2010; Del Pozo, 2018; Law and Tan, 2022). The biofilm-associated infections can subsequently induce chronic infections, resulting in a considerable burden on the global healthcare system (Hall-Stoodley et al., 2004; Høiby et al., 2015). However, there is limited research regarding the clinical implications of biofilm formation. A previous study demonstrated that those infected with biofilm-forming isolates of *A. baumannii* had a probable history of ICU admission, use of antibiotics, and lesser severity of disease (Zhang et al., 2016). The study did not demonstrate an influence of biofilm formation on the clinical outcomes in the patients. A single-institute study documented that the mortality during an initial infection was significantly more common in patients with the biofilm-forming isolates, compared with those with the non-biofilm-forming isolates (Barsoumian et al., 2015). However, the low attributable mortality (7.1%) among the study population made it difficult to draw any conclusions regarding the clinical outcomes of biofilm formation ability of *A. baumannii* (Barsoumian et al., 2015). Recently, in a cohort study involving 273 patients, we found that biofilm formation was not associated with worse outcomes in patients with *A. baumannii* bacteremic pneumonia (Wang et al., 2018). As *A. baumannii* contributes to a variety of biofilm-associated infections, we included all types of infections to delineate the effects of biofilm formation on the clinical outcomes in this study. We found that biofilm formation capability was not an independent risk factor of 14-day and 28-day mortality in patients with *A. baumannii* bacteremia.

Bacterial cells embedded in the biofilms are known to be resistant to antimicrobials through several mechanisms, including limited penetration of the antimicrobials, slow growth rate of the bacterial cells in biofilms, physiological heterogeneity of the biofilms, and the expression of some resistance genes (Lewis, 2001; Mah and O’Toole, 2001; Harrison et al., 2007; Olsen, 2015). These conditions make biofilms difficult to eradicate and therefore, the correlation between resistance to individual antibiotics and biofilm formation remains elusive. While some studies have shown a positive correlation between resistance to individual antibiotics and biofilm formation (Rao et al., 2008; Badave and Kulkarni, 2015), others have found a negative correlation between the biofilm formation ability and carbapenem resistance (Rodríguez-Baño et al., 2008; Qi et al., 2016; Wang et al., 2018). Our previous studies have also demonstrated that most carbapenem-resistant *A. baumannii* transformants exhibit reduced biofilm-forming abilities (Wang et al., 2018). Our current study has shown similar findings, as the biofilm-forming isolates exhibited lower rates of carbapenem-resistance than the non-biofilm-forming isolates (56.7% *vs.* 59.3%). These findings suggest that the complexity of biofilm composition, and not of the bacteria themselves in the biofilms, may contribute to the antibiotic resistance of the biofilms (Lewis, 2001; Mah and O’Toole, 2001; Harrison et al., 2007; Olsen, 2015). Further studies are needed to elucidate the detailed mechanisms of antimicrobial resistance in the biofilms.

A major strength of this study was the larger sample size of patients who were enrolled from multiple medical centers. In order to exclude colonized isolates, all the bacterial strains collected in this study were isolated from blood samples. We enrolled patients with *A. baumannii* bacteremia caused by different sources of infection to represent the real-world clinical situations. Another strength was the use of multivariate analysis to delineate the risk factors for mortality in patients with bacteremia caused by biofilm-forming isolates of *A. baumannii*.

This retrospective study had several limitations, including selection bias and inconsistencies in patient care among the different hospitals. Another key limitation is that the *in vitro* formation of biofilms does not represent the actual *in vivo* conditions. Although there are several methods for the detecting *in vitro* biofilm formation, there is currently no gold-standard protocol for its quantification. Furthermore, it is challenging to assess the biofilm formation inside the human body. In addition, the *in vitro* conditions may be quite different from those of the human environment.

In conclusion, this is the first large sample size study on the clinical outcomes of patients with *A. baumannii* bacteremia. Our results demonstrated that the biofilm-forming ability was not an independent risk factor for mortality in patients with *A. baumannii* bacteremia. Patients with *A. baumannii* bacteremia with a greater severity of disease, who were infected with carbapenem-resistant isolates, or had received an inappropriate antimicrobial therapy had worse outcomes. Patients with congestive heart failure were more likely to be infected with biofilm-forming isolates, while those with an arterial line were less likely to be infected with them. Among the patients infected with biofilm-forming isolates, those with hematological malignancies, infected with carbapenem-resistant isolates, and those with greater severity of disease (higher APACHE II scores) were associated with worse clinical outcomes. Further studies are required to establish the optimal treatment for bacteremia caused by the biofilm-forming isolates of *A. baumannii*.

## Data availability statement

The raw data supporting the conclusions of this article will be made available by the authors, without undue reservation.

## Ethics statement

The studies involving human participants were reviewed and approved by Changhua Christian Hospital (140514) Mackay Memorial Hospital (14MMHIS125) National Taiwan University Hospital (201008047R) Taipei Veterans General Hospital (2015-04-003C) Tri-Service General Hospital (1-103-05-100). Written informed consent for participation was not required for this study in accordance with the national legislation and the institutional requirements.

## Author contributions

Conceptualization, T-TC, Y-TL, and Y-CW; Data curation, AC, C-PL, Y-ML, and Y-SY; Formal analysis, T-TC, T-WH, and J-RS; Funding acquisition, Y-TL and Y-CW; Investigation, S-CK, AC, and C-PL; Methodology, T-TC, Y-TL, and Y-CW; Project administration, Y-TL, and Y-CW; Resources, Y-ML, Y-SY, and T-LC; Software, T-WH, J-RS, and S-CK; Supervision, T-LC, Y-TL, and Y-CW; Validation, T-LC, Y-TL, and Y-CW; Visualization, C-PL, Y-ML, and Y-SY; Writing – original draft, T-TC, Y-TL, and Y-CW; Writing – review and editing, T-TC, Y-TL, and Y-CW. All authors contributed to the article and approved the submitted version.

## Funding

This work was supported by grants from Taipei Veterans General Hospital [V108C-012, VTA108-T-2-3, VTA109-T-3-2, VTA110-V4-5-2, VTA111-T-3-3], Tri-Service General Hospital [TSGH-E-109237, TSGH-E-110205, TSGH-E-111245], and the Ministry of Science and Technology [MOST-108-2314-B-016 -029, MOST-109-2314-B-016 -056, MOST-110-2314-B-016-063, MOST-110-2314-B-075-072, MOST 108-2314-B-075-034-MY3, MOST 107-2314-B-075-066-MY3].

## Conflict of interest

The authors declare that the research was conducted in the absence of any commercial or financial relationships that could be construed as a potential conflict of interest.

## Publisher’s note

All claims expressed in this article are solely those of the authors and do not necessarily represent those of their affiliated organizations, or those of the publisher, the editors and the reviewers. Any product that may be evaluated in this article, or claim that may be made by its manufacturer, is not guaranteed or endorsed by the publisher.
