# Supplementary material for: Biofilm formation is not an independent risk factor for mortality in patients with Acinetobacter baumannii bacteremia
Source: Front Cell Infect Microbiol. 2022 Sep 16;12:964539. doi: 10.3389/fcimb.2022.964539 (PMC9523115; doi:10.3389/fcimb.2022.964539)
Supplement: Supplementary file 1 [file Table_1.docx]

Supplementary Table S1. Clinical characteristics of patients with bacteremia caused by biofilm-forming *Acinetobacter baumannii* who survived or died within 28 days of bacteremia onset.

|  | Survival  (n = 148) | Non-survival  (n = 104) | *P* |
| --- | --- | --- | --- |
| Demographic characteristics |  |  |  |
| Age, median (IQR), years | 72 (60–81) | 68.5 (54–81) | 0.130 |
| Male sex, No. (%) | 97 (65.5) | 74 (71.2) | 0.411 |
| Acquired in ICU, No. (%) | 47 (31.8) | 54 (51.9) | 0.002 |
| Length of hospitalization before  bacteremia, median (IQR), days | 14 (4–33.5) | 18 (9–37) | 0.097 |
| Previous use of antibiotics, No. (%) |  |  |  |
| Aminoglycosides | 12 (8.1) | 8 (7.7) | 1.000 |
| Penicillins | 12 (8.1) | 9 (8.7) | 1.000 |
| β-lactam/β-lactamase inhibitors  (except sulbactam) | 15 (10.1) | 17 (16.3) | 0.179 |
| Sulbactam | 8 (5.4) | 3 (2.9) | 0.533 |
| Non-anti-pseudomonas Cephalosporins | 28 (18.9) | 18 (17.3) | 0.869 |
| Anti-pseudomonas Cephalosporins | 27 (18.2) | 25 (24.0) | 0.273 |
| Group 2 Carbapenems | 18 (12.2) | 20 (19.2) | 0.153 |
| Fluoroquinolones | 15 (10.1) | 24 (23.1) | 0.007 |
| Tigecycline | 5 (3.4) | 7 (6.7) | 0.242 |
| Colistin | 1 (0.7) | 5 (4.8) | 0.084 |
| Teicoplanin | 17 (11.5) | 19 (18.3) | 0.146 |
| Fluconazole | 9 (6.1) | 13 (12.5) | 0.111 |
| Comorbid condition, No. (%) |  |  |  |
| Liver cirrhosis | 12 (8.1) | 15 (14.4) | 0.147 |
| Chronic obstructive pulmonary disease | 25 (16.9) | 15 (14.4) | 0.727 |
| Chronic kidney disease | 42 (28.4) | 42 (40.4) | 0.057 |
| Type 2 diabetes mellitus | 50 (33.8) | 24 (23.1) | 0.070 |
| Hypertension | 58 (39.2) | 29 (27.9) | 0.080 |
| Coronary artery disease | 14 (9.5) | 14 (13.5) | 0.416 |
| Congestive heart failure | 25 (16.9) | 23 (22.1) | 0.330 |
| Cerebrovascular accident | 35 (23.6) | 15 (14.4) | 0.079 |
| Collagen vascular disease | 1 (0.7) | 13 (12.5) | <0.001 |
| Immunosuppressant therapy | 26 (17.6) | 35 (33.7) | 0.004 |
| Solid tumor | 33 (22.3) | 24 (23.1) | 0.880 |
| Hematological malignancy | 9 (6.1) | 17 (16.3) | 0.011 |
| Chemotherapy | 14 (9.5) | 18 (17.3) | 0.083 |
| Recent surgery | 37 (25.0) | 11 (10.6) | 0.005 |
| Previous ICU admission | 64 (43.2) | 62 (59.6) | 0.015 |
| Charlson comorbidity index,  median (IQR) | 2 (1–5) | 2 (1–4.75) | 0.701 |
| Invasive Procedures, No. (%) |  |  |  |
| Central venous catheter | 36 (24.3) | 35 (33.7) | 0.119 |
| Arterial line | 14 (9.5) | 10 (9.6) | 1.000 |
| Tracheostomy | 13 (8.8) | 9 (8.7) | 1.000 |
| Ventilator use | 57 (38.5) | 54 (51.9) | 0.035 |
| Hemodialysis | 10 (6.8) | 10 (9.6) | 0.480 |
| Thoracic drain | 1 (0.7) | 4 (3.8) | 0.163 |
| Abdominal drain | 6 (4.1) | 7 (6.7) | 0.393 |
| Total parental nutrition | 9 (6.1) | 8 (7.7) | 0.620 |
| Carbapenem resistance, No. (%) | 63 (42.6) | 80 (76.9) | <0.001 |
| Appropriate antimicrobial therapy, No. (%) | 59 (39.9) | 28 (26.9) | 0.043 |
| Outcome |  |  |  |
| Shock, No. (%) | 27 (18.2) | 54 (51.9) | <0.001 |
| APACHE II score, median (IQR) | 15 (12–22) | 29 (21.25–36.75) | <0.001 |
| Sources of bacteremia, No. (%) |  |  |  |
| Pneumonia | 48 (32.4) | 63 (60.6) | <0.001 |
| Catheter related bloodstream infection | 28 (18.9) | 17 (16.3) | 0.621 |
| Urinary tract infection | 20 (13.5) | 5 (4.8) | 0.031 |
| Intra-abdominal infection | 17 (11.5) | 10 (9.6) | 0.684 |
| Skin and soft tissue infection | 7 (4.7) | 4 (3.8) | 1.000 |
| Surgical Site Infection | 1 (0.7) | 1 (1.0) | 1.000 |
| Primary bacteremia | 35 (23.6) | 21 (20.2) | 0.542 |
| Central nerve system | 0 (0.0) | 0 (0.0) | 1.000 |
| Multisite infection | 6 (4.1) | 13 (12.5) | 0.015 |
| Other infection sites | 15 (10.1) | 6 (5.8) | 0.254 |

Note. IQR, interquartile range; ICU, intensive care unit; APACHE II, Acute Physiology and Chronic Health Evaluation II.

Supplementary Table S2. Logistic regression analysis for the risk of 28-day mortality in patients with bacteremia caused by biofilm-forming *Acinetobacter baumannii*.

|  | Crude model OR (95% CI) | *P* | Mode1 1^*^  OR (95% CI) | *P* |
| --- | --- | --- | --- | --- |
| Acquired in ICU | 2.321  (1.383–3.894) | 0.001 | 1.085  (0.407–2.891) | 0.870 |
| Previous use of Fluoroquinolones | 2.660  (1.318–5.368) | 0.006 | 1.119  (0.406–3.078) | 0.828 |
| Collagen vascular disease | 21  (2.702–163.237) | 0.004 | 8.266  (0.758–90.084) | 0.083 |
| Immunosuppressant therapy | 2.380  (1.323–4.281) | 0.004 | 1.062  (0.430–2.627) | 0.896 |
| Hematological malignancy | 3.018  (1.288–7.069) | 0.011 | 3.636  (1.011–13.072) | 0.048 |
| Recent surgery | 0.355  (0.171–0.734) | 0.005 | 0.505  (0.187–1.364) | 0.178 |
| Previous ICU admission | 1.937  (1.165–3.224) | 0.011 | 0.676  (0.266–1.715) | 0.410 |
| Ventilator use | 1.724  (1.038–2.864) | 0.035 | 2.277  (0.885–5.858) | 0.088 |
| Carbapenem resistance | 4.497  (2.567–7.878) | <0.001 | 2.945  (1.344–6.453) | 0.007 |
| Appropriate antimicrobial therapy | 0.556  (0.323–0.958) | 0.034 | 0.522  (0.241–1.133) | 0.100 |
| Shock | 4.840  (2.744–8.537) | <0.001 | 1.829  (0.814–4.113) | 0.144 |
| APACHE II score | 1.150  (1.109–1.193) | <0.001 | 1.151  (1.098–1.206) | <0.001 |
| Pneumonia | 3.201  (1.898–5.399) | <0.001 | 1.677  (0.734–3.836) | 0.220 |
| Urinary tract infection | 0.323  (0.117–0.891) | 0.029 | 0.328  (0.074–1.457) | 0.143 |
| Multisite infection | 3.381  (1.241–9.213) | 0.017 | 1.161  (0.292–4.607) | 0.832 |

Note. ICU, intensive care unit; APACHE II, Acute Physiology and Chronic Health Evaluation II.

^*^Adjusted by all factors included in the table.
